# Cost-effectiveness of Axicabtagene Ciloleucel and Tisagenlecleucel as Second-line or Later Therapy in Relapsed or Refractory Diffuse Large B-Cell Lymphoma

**DOI:** 10.1001/jamanetworkopen.2022.45956

**Published:** 2022-12-15

**Authors:** Jee H. Choe, Hisham Abdel-Azim, William V. Padula, Mohamed Abou-el-Enein

**Affiliations:** 1 Department of Pharmaceutical and Health Economics, School of Pharmacy, University of Southern California, Los Angeles; 2 Leonard D. Schaeffer Center for Health Policy & Economics, University of Southern California, Los Angeles; 3 Loma Linda University School of Medicine, Cancer Center, Children Hospital and Medical Center, Loma Linda, California; 4 Division of Medical Oncology, Department of Medicine, Keck School of Medicine, University of Southern California, Los Angeles; 5 Department of Stem Cell Biology and Regenerative Medicine, Keck School of Medicine, University of Southern California, Los Angeles; 6 USC/CHLA Cell Therapy Program, University of Southern California and Children’s Hospital of Los Angeles, Los Angeles

## Abstract

**Importance:**

Chimeric antigen receptor (CAR) T cell therapies are approved as a third-line or later therapy for several hematological malignant neoplasms. Recently, randomized clinical trials have investigated their efficacy as a second-line treatment in high-risk relapsed or refractory diffuse large B-cell lymphoma (DLBCL) compared with salvage chemotherapy followed by hematopoietic stem cell transplantation (HSCT).

**Objective:**

To evaluate the cost-effectiveness of axicabtagene ciloleucel and tisagenlecleucel vs standard care (SC) as second-line or later therapy for relapsed or refractory DLBCL, from both US health care sector and societal perspectives at a cost-effectiveness threshold of $150 000 per quality-adjusted life-year (QALY).

**Design, Setting, and Participants:**

This economic evaluation assessed cost-effectiveness using a partitioned survival model with 2021 US dollars and QALYs over a lifetime horizon. Model inputs were derived from 2 randomized clinical trials (ZUMA-7 and BELINDA) and published literature. In the trials, patients who did not respond to SC received CAR T cells (treatment switching or crossover), either outside the protocol (ZUMA-7) or as part of the protocol (BELINDA). A separate scenario analysis compared second-line axicabtagene ciloleucel with SC alone without treatment crossover to CAR T cell therapy. Data analysis was performed from December 18, 2021, to September 13, 2022.

**Exposures:**

CAR T cell therapy (axicabtagene ciloleucel and tisagenlecleucel) compared with salvage chemotherapy followed by HSCT.

**Main Outcomes and Measures:**

Costs and QALYs were used to derive incremental cost-effectiveness ratios (ICERs) for the health care sector and societal perspectives. Cost and QALYs were discounted at 3.0% annually. Univariate and multivariate probabilistic sensitivity analysis using 10 000 Monte Carlo simulations were applied to test model uncertainty on the ICER.

**Results:**

Second-line axicabtagene ciloleucel was associated with an ICER of $99 101 per QALY from the health care sector perspective and an ICER of $97 977 per QALY from the societal perspective, while second-line tisagenlecleucel was dominated by SC (incremental costs of $37 803 from the health care sector and $39 480 from the societal perspective with decremental QALY of -0.02). Third-line or later tisagenlecleucel was associated with an ICER of $126 593 per QALY from the health care sector perspective and an ICER of $128 012 per QALY from the societal perspective. Based on the scenario analysis of no treatment switching, second-line axicabtagene ciloleucel yielded an ICER of $216 790 per QALY from the health care sector perspective and an ICER of $218 907 per QALY from the societal perspective, compared with SC. When accounting for patients achieving prolonged progression-free survival who would not incur progression-related costs, in this scenario ICER changed to $125 962 per QALY from the health care sector perspective and $122 931 per QALY from the societal perspective. These results were most sensitive to increased list prices of CAR T cell therapy and QALY losses associated with axicabtagene ciloleucel and tisagenlecleucel.

**Conclusions and Relevance:**

These findings suggest that second-line axicabtagene ciloleucel and third-line or later tisagenlecleucel were cost-effective in treating patients with relapsed or refractory DLBCL at the cost-effectiveness threshold of $150 000 per QALY. However, uncertainty remains regarding the best candidates who would experience value gains from receiving CAR T cell therapy.

## Key Points

**Question:**

Compared with standard care (SC), are anti-CD19 chimeric antigen receptor (CAR) T cell therapies cost-effective for treating B-cell lymphomas in the second-line or above settings?

**Findings:**

This economic evaluation using data from published literature found that second-line axicabtagene ciloleucel was associated with an incremental cost-effectiveness ratio of $99 101 per quality-adjusted life-year (QALY) from a US health sector perspective and $97 977 per QALY from a societal perspective, while third-line or later tisagenlecleucel had an incremental cost-effectiveness ratio of $126 593 per QALY from a health sector perspective and $128 012 per QALY from a societal perspective.

**Meaning:**

These findings suggest that axicabtagene ciloleucel was cost-effective as second-line therapy while tisagenlecleucel was cost-effective only as third-line or later therapy in treating patients with relapsed or refractory diffuse large B-cell lymphoma at the willingness-to-pay threshold of $150 000 per QALY.

## Teaser

This economic evaluation assesses the cost-effectiveness of axicabtagene ciloleucel and tisagenlecleucel vs standard care as second-line or later therapy for relapsed or refractory diffuse large B-cell lymphoma.

## Introduction

Diffuse large B-cell lymphoma (DLBCL) is the most common non-Hodgkin lymphoma subtype in the United States and worldwide.^1^ Patients with DLBCL are commonly treated with chemoimmunotherapy, and approximately 75% to 80% of these patients achieve long-term remission.^2^ Approximately 30% to 40% of patients who respond to salvage chemotherapy continue consolidation treatment with second-line hematopoietic stem cell transplantation (HSCT).^3^ Nevertheless, suboptimal outcomes persist, as approximately half of these patients ultimately relapse.^3^

Chimeric antigen receptor (CAR) T cell therapies, such as axicabtagene ciloleucel and tisagenlecleucel, have become commercially available as novel therapeutic approaches for patients with B cell malignant neoplasms who are refractory or relapsed after 2 or more lines of standard therapy. The premise of CAR T cells is to genetically modify patients’ autologous T cells to identify specific targets on cancer cells and eliminate them. Although CAR T cell therapies have demonstrated improved overall response rates,^4,5,6^ concerns remain regarding clinical trial designs based on single groups, the lack of longer-term outcome follow-ups, and the cost-effectiveness of CAR T cell therapies, with high list prices ranging from $373 000 to $410 300 per treatment.^7^

Previous studies assessed the cost-effectiveness of CAR T cell therapy in treating patients with relapsed or refractory DLBCL as a third-line or later therapy from the US health care sector perspective. Third-line or later axicabtagene ciloleucel was associated with incremental survival gains, ranging from 1.5 to 7.7 quality-adjusted life-years (QALY), at incremental cost-effectiveness ratios (ICERs) ranging from $58 146 to $230 900 per QALY.^8,9,10,11^ Similarly, tisagenlecleucel was associated with the incremental survival gains of 3.9 QALYs at an ICER of $168 000 per QALY when assuming a 35% 5-year progression-free survival (PFS).^8^ Such wide variation in ICER results reflected uncertainty in longer-term efficacy outcomes and limitations of the single-group trial design. Thus, the gap in current knowledge on the cost-effectiveness of CAR T cell therapy highlights a need to account for these shortcomings.

Recently, results from ongoing multicenter randomized clinical trials investigating axicabtagene ciloleucel (ZUMA-7: NCT03391466)^12^ and tisagenlecleucel (BELINDA: NCT03570892)^13^ were published comparing CAR T cell therapy with salvage chemotherapy followed by HSCT as second-line therapy for the treatment of relapsed or refractory DLBCL. ZUMA-7 demonstrated remarkable complete response in 65% of patients in the axicabtagene ciloleucel group compared with 32% of patients in the standard care (SC) group. In BELINDA, tisagenlecleucel was not superior to SC, with a reported complete response in 28.4% of patients in the tisagenlecleucel group compared with 27.5% of patients in the SC group. This resulted in the US Food and Drug Administration expanding the label for axicabtagene ciloleucel to be offered as second-line treatment for adults with relapsed or refractory DLBCL. In addition, updated results have been published^14^ from the ongoing multicenter, single-group JULIET trial (NCT02445248), with a median follow-up of 40 months for tisagenlecleucel as a third-line therapy. These clinical reports have influenced the adoption of CAR T cell therapy in clinical practice and may have implications on reimbursement policies and health insurance coverage. In this study, we conducted several cost-effectiveness analyses on CAR T cell therapy vs SC as second-line or later therapy in treating patients with relapsed or refractory DLBCL from both the US health care sector and societal perspectives.

## Methods

This economic evaluation was deemed exempt from review and informed consent by the University of Southern California because it is not considered human participants research. This study is reported following the Consolidated Health Economic Evaluation Reporting Standards (CHEERS) reporting guideline.

### Treatment Strategies

We constructed a cost-effectiveness model for each treatment strategy by comparing axicabtagene ciloleucel and tisagenlecleucel independently with salvage high-dose chemotherapy and consolidative autologous HSCT as the second-line therapy. We then compared tisagenlecleucel with salvage chemotherapy for refractory disease as the third-line or later therapy. Treatment strategies were derived from ZUMA-7 for second-line axicabtagene ciloleucel, BELINDA for second-line tisagenlecleucel, and JULIET for tisagenlecleucel as the third-line or later treatment (eMethods in Supplement 1).^12,13,14^

After CAR T cell infusion, patients may proceed to autologous HSCT (auto-HSCT) or allogeneic HSCT (allo-HSCT). ZUMA-7 reported the proportion of patients receiving a subsequent HSCT (3.3% of patients receiving auto-HSCT and 0.6% of patients receiving allo-HSCT in the axicabtagene ciloleucel group; 2.8% of patients receiving auto-HSCT in the SC group).^15^ In addition, JULIET reported subsequent allo-HSCT in 6% of patients and auto-HSCT in 0.8% of patients receiving tisagenlecleucel.^16^ The model estimated the total treatment costs by adding HSCT costs to either the second-line axicabtagene ciloleucel cohort or third-line tisagenlecleucel cohort according to the reported outcomes. Since there were no data reported for patients who received second-line tisagenlecleucel receiving subsequent HSCT, this cost component was not included in the model for this cohort.

The SC second-line treatment for patients with relapsed or refractory DLBCL that is commonly used in clinical trials includes salvage chemotherapy followed by HSCT.^12,13^ Since JULIET was a single-group trial, we used patients from the SCHOLAR-1 study^3^ as the reference for the SC group in the third-line or later settings. Despite potential differences in patient populations and study design in SCHOLAR-1, it was the largest patient-level pooled analysis that included a US population and was identified as the most appropriate comparator for third-line or later CAR T cell therapy.^17^

A total of 56% of SC patients in ZUMA-7 and 50% of SC patients in BELINDA ultimately received CAR T cell therapy as a third-line treatment.^12,13^ In ZUMA-7, it was reported that the SC overall survival (OS) may have been confounded by this crossover. Therefore, we established a scenario analysis to compare axicabtagene ciloleucel with salvage chemotherapy followed by HSCT only. We used the rank-preserving structural failure time method to adjust for treatment switching.^12^ Since BELINDA did not report the confounding associated with CAR T cell therapy switching, the scenario analysis was not conducted for second-line tisagenlecleucel.

### Model Structure

**Figure 1.**
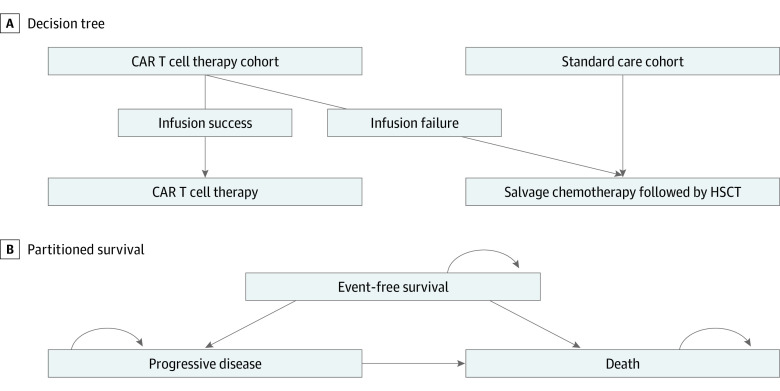
Hybrid Decision Tree and 3-State Partitioned Survival Model of Chimeric Antigen Receptor (CAR) T Cell Therapy vs Standard Care Treatment Strategies Once patients entered partitioned survival model’s state after the decision tree model, they either remained in the same state or progressed to later states. HSCT indicates hematopoietic stem cell transplantation.

Partitioned survival models were constructed to include 3 states: event-free survival (EFS) for second-line CAR T cell therapy or PFS for third-line or later CAR T cell therapy, progressive disease (PD), and death. The models compared the cost-effectiveness of CAR T cell therapy against SC from the US health care sector and societal perspectives (Figure 1). If patients could not proceed to CAR T cell infusion due to manufacturing failure, those patients continued the SC treatment.^12,13,14^ Using Excel spreadsheet software version 16.66.1 (Microsoft), all models were constructed with a 1-month cycle over the lifetime horizon. We followed the methodological guidelines set by the US Second Panel on Cost-Effectiveness in Health and Medicine.^18^ All costs were adjusted to 2021 US dollars. Costs and QALYs were discounted at 3% annually.^19^

### Model Parameters

Transitional probabilities for the partitioned states were estimated using parametric survival functions derived from OS and EFS (or PFS) curves from ZUMA-7, BELINDA, JULIET, and SCHOLAR-1 (eFigure 1, eFigure 2, and eTable 1 in Supplement 1). Since SCHOLAR-1 did not report the PFS curve, we used the OS curve to derive the PFS, assuming a constant hazard ratio of 0.7 over time. After 40 months (the longest follow-up period reported for pivotal trials of CAR T cell therapies^14^), patients who remained in the EFS or PFS state were assumed to achieve the long-term DLBCL survivals reported in the literature.^20^

Preference-weighted utilities for all models were assigned to EFS or PFS and PD states based on the Norwegian Medicines Agency single technology assessment, while accounting for disutility associated with treatment duration. These utility values were derived from health survey questionnaire data reported in the JULIET trial. Disutility represents the decrement in utility (valued quality of life) due to a condition.^21^ We assigned 1-cycle disutility to CAR T cell therapies and 3-cycle disutility to salvage chemotherapy and HSCT as indicated in the Norwegian Medicines Agency single technology assessment.^21^

Formal health care–associated costs were included in the US health care sector and societal perspectives,^7,8,9,12,13,14,15,16,20,21,22,23,24,25,26,27,28,29,30,31,32,33,34,35,36^ and informal health care costs were included in the US societal perspective only (Table 1). Health care cost estimates were extracted from the literature and mostly based on Centers for Medicare & Medicaid Services (CMS) reimbursement schedule. CAR T cell treatment costs were modeled according to observed hospitalization data from the JULIET trial as reported in Yang et al.^22^ Inpatient and ICU costs were calculated separately.^23^ Adverse event costs were estimated based on grade 3 or greater adverse events with incidence rates greater or equal to 5%, including cytokine release syndrome and neurotoxic effects (eTable 2 in Supplement 1). CAR T cell therapy travel-related out-of-pocket costs were reported to be different across settings and thus were included as informal costs.^24,25^ Costs were estimated based on 3 settings: academic hospitals, a combination of academic and community multispecialty hospitals, or any specialty centers.^24^ Our base cases for the partitioned survival models used the combination of academic and community multispecialty hospitals.

**Table 1.** Model Parameters

Parameter	Base case inputs (PSA range)	Distribution	Source
**Clinical inputs**			
CAR T cell therapy infusion rate			
Axicabtagene ciloleucel (2L)	0.94 (0.37 to 1.00)	β	Locke et al,^12^ 2022
Tisagenlecleucel			
2L	0.96 (0.32 to 1.00)	β	Bishop et al,^13^ 2022
≥3L	0.69 (0.38 to 0.94)	β	Schuster et al,^14^ 2021
Standard mortality rate	1.09 (0.29 to 2.52)		Maurer et al,^20^ 2014
Utility			
EFS or PFS	0.83 (0.44 to 0.98)	β	NOMA^21^
PD	0.71 (0.44 to 0.91)	β	NOMA^21^
Disutility: treatment	−0.15 (−0.23 to −0.09)	β	NOMA^21^
**Direct costs, $** ^a^			
CAR T cell therapy			
Lymphodepleting therapy	3172 (1736 to 5169)	γ	Yang et al,^22^ 2020
EFS and PFS follow-up, y			
1	510 (277 to 840)	γ	Qi et al,^16^ 2021
2	156 (83 to 248)	γ	Qi et al,^16^ 2021
3-5	93 (47 to 146)	γ	Qi et al,^16^ 2021
>5	12 (7 to19)	γ	Qi et al,^16^ 2021
Axicabtagene ciloleucel			
Bridging therapy	633 (363 to 949)	γ	Locke et al,^12^ 2022
			CMS^26^
Axicabtagene ciloleucel list price	399 000		RedBook^7^
Pretreatment costs^b^	2731 (1578 to 4187)	γ	Yang et al,^22^ 2020
Infusion costs^c^	512 (293 to 783)	γ	Yang et al,^22^ 2020, CMS^27^
Inpatient and ICU costs	75 849 (40 928 to 115 108)	γ	Liu et al,^23^ 2021
Follow-up costs after infusion	4222 (2468 to 6362)	γ	Yang et al,^22^ 2020
Rate of patients receiving IVIG	0.47 (0.22 to 0.71)	β	Locke et al,^12^ 2022
B-cell aplasia (mean 12 IVIG infusions)	38 500 (23 544 to 58 702)	γ	Liu et al,^23^ 2021
			Yang et al,^22^ 2020
AE management (grade ≥3)	27 326 (16 599 to 42 007)	γ	Yang et al,^22^ 2020
			Roth et al,^9^ 2018
			Broder et al,^28^ 2020
			HCUP^29^
Rate			
Allo-HSCT	0.03 (0.02 to 0.05)	γ	Locke et al,^15^ 2022
Auto-HSCT	0.01 (<0.01 to 0.01)	γ	Locke et al,^15^ 2022
Tisagenlecleucel			
Bridging therapy			
2L	9049 (5385 to 13 843)	γ	Bishop et al,^13^ 2022
			CMS^26,30^
≥3L	2685 (1161 to 3731)	γ	Sesques et al,^31^ 2020
			CMS^26,30^
Tisagenlecleucel list price	373 000		RedBook^7^
Pretreatment costs^d^	9675 (5345 to 14 695)	γ	Yang et al,^22^ 2020
Infusion costs^e^	1455 (825 to 2190)	γ	Yang et al,^22^ 2020
			CMS^27^
Follow-up costs after infusion	21 383 (10 885 to 32 163)	γ	Yang et al,^22^ 2020
Rate of patients receiving IVIG	0.18 (0.11 to 0.29)	β	Yang et al,^22^ 2020
B-cell aplasia (mean 1.8 IVIG infusions)	4583 (2673 to 7032)	γ	Yang et al,^22^ 2020
AE management, grade ≥3			
2L	25 705 (14 997 to 40 179)	γ	Yang et al,^22^ 2020
			Roth et al,^9^ 2018
			HCUP^29^
≥3L	31 022 (16 949 to 49 941)	γ	Yang et al,^22^ 2020
Rate, ≥3L Tisagenlecleucel			
Allo-HSCT	0.06 (0.04 to 0.10)	β	Qi et al,^16^ 2021
Auto-HSCT	0.01 (<0.01 to 0.01)	β	Qi et al,^16^ 2021
**Salvage chemotherapy costs, $** ^a^			
Salvage chemo drug costs			
2L vs Axicabtagene ciloleucel	29 433 (17 827 to 48 399)	γ	Locke et al,^12^ 2022
			CMS^26^
≥2L vs Tisagenlecleucel	35 088 (21 002 to 55 901)	γ	Bishop et al,^13^ 2022
			CMS^26^
Administrative costs^f^	3666 (1878 to 5814)	γ	Huntington et al,^32^ 2018
AE management, grade ≥3			
2L vs Axicabtagene ciloleucel	22 981 (13 661 to 34 235)	γ	Yang et al,^22^ 2020
			Roth et al,^9^ 2018
			Broder et al,^28^ 2017
			HCUP^29^
2L vs Tisagenlecleucel	32 734 (19 348 to 55 205)	γ	Yang et al,^20^ 2020
			Roth et al,^9^ 2018
			HCUP^29^
≥3L	7607 (5005 to 10 430)	γ	Crump et al,^26^ 2014
			Yang et al,^20^ 2020,
			Lin et al,^8^ 2019
			HCUP^29^
PFS follow-up, y			
1	228 (131 to 354)	γ	Qi et al,^16^ 2021
2	168 (88 to 262)	γ	Qi et al,^16^ 2021
3-5	49 (28 to 75)	γ	Qi et al,^16^ 2021
>5	12 (7 to19)	γ	Qi et al,^16^ 2021
Rate			
Auto-HSCT (2L vs Axicabtagene ciloleucel)	0.38 (0.20 to 0.57)	β	Locke et al,^12^ 2022
Auto-HSCT (2L vs Tisagenlecleucel)	0.33 (0.17 to 0.50)	β	Bishop et al,^13^ 2022
Allo-HSCT (≥3L)	0.11 (0.06 to 0.17)	β	Qi et al,^16^ 2021
Auto-HSCT (≥3L)	0.30 (0.17 to 0.48)	β	Crump et al,^33^ 2014
Crossover to CAR T cell therapy			
2L vs Axicabtagene ciloleucel	0.56 (0.31 to 0.81)	β	Locke et al,^12^ 2022
2L vs Tisagenlecleucel	0.51 (0.27 to 0.71)	β	Bishop et al,^13^ 2022
Common costs to CAR T cell therapy and standard care			
Allo-HSCT	337 809 (192 634 to 519 843)	γ	Godara et al,^34^ 2021
Auto-HSCT (1-y follow-up)	240 925 (110 579 to 382 473)	γ	Broder et al,^35^ 2017
Progression-related costs	2864 (1395 to 4091)	γ	Huntington et al,^32^ 2018
End-of-life health care sector costs	18 076 (0 to 70 165)	γ	Chastek et al,^36^ 2012
**Indirect costs, $** ^a^			
Travel-related costs			
CAR T by type of treatment center			
Academic	5367 (3244 to 8589)	γ	Snyder et al,^24^ 2021
Academic + community	4512 (2381 to 7212)	γ	Snyder et al,^24^ 2021
Any specialty	3738 (2197 to 5504)	γ	Snyder et al,^24^ 2021
Salvage chemotherapy			
Patient time	1687 (1304 to 2171)	γ	Sarkar et al,^25^ 2019
Caregiver	577 (11 to 2802)	γ	Sarkar et al,^25^ 2019
Parking, meals, and transportation	315 (140 to 598)	γ	Sarkar et al,^25^ 2019

Abbreviations: AE, adverse event; allo, allogeneic; auto, autologous; CAR, chimeric antigen receptor; CMS, Centers for Medicare & Medicaid Services; EFS, event-free survival; HCUP, Healthcare Cost and Utilization Project; HSCT, hematopoietic stem cell transplant; ICU, intensive care units; IVIG, intravenous immunoglobulin; L, line of therapy setting; NOMA, Norwegian Medicines Agency; PD, progressive disease; PFS, progression-free survival; PSA, probability sensitivity analysis.

^a^ Costs are provided in adjusted 2021 US dollars.

^b^ Includes laboratory and procedure test and professional visit costs.

^c^ Includes administration, laboratory and procedure test, and professional visit costs.

^d^ Includes inpatient, ICU, laboratory and procedure test, and professional visit costs.

^e^ Includes administration, inpatient, ICU, laboratory and procedure test, and professional visit costs.

^f^ Includes inpatient, outpatient, and emergency department visit costs.

### Sensitivity Analysis

We conducted univariate sensitivity analyses to assess parametric uncertainties by varying all parameters within 25%. In addition, scenario analyses were conducted on the following conditions: (1) confounding caused by crossover from SC to CAR T cell therapy, (2) type of the treatment center authorized to administer CAR T cell therapy, and (3) costs associated with patients who may achieve a PFS state after third-line crossover CAR T cell therapy. We estimated the PFS and progression-related costs of third-line CAR T cell therapy for patients based on the US Lymphoma CAR T Consortium study.^37^ We assumed that the utility of these patients is the same as extrapolated from the Norwegian Medicines Agency study.^21^ We also conducted a bayesian multivariate probabilistic sensitivity analysis with 10 000 Monte Carlo simulations to account for uncertainty in cost-effectiveness calculations. Lastly, we analyzed the expected value of perfect information to reduce uncertainty in allocating CAR T cell therapies to the appropriate patients who may benefit in the most cost-effective manner. We estimated the expected value of perfect information using 10 000 variations of all model parameters. All the sensitivity analyses were conducted from the US societal perspective. Data analysis was performed from December 18, 2021, to September 13, 2022.

## Results

### Second-line CAR T Cell Therapy

The second-line axicabtagene ciloleucel cohort experienced incremental survival benefits of 0.60 QALYs compared with the SC group (Table 2). The axicabtagene ciloleucel group was associated with incremental costs of $59 754 from the US health care sector perspective and $59 076 from the societal perspective, yielding ICERs of $99 101 per QALY from the US health care sector perspective and $97 977 per QALY from the societal perspective. However, with respect to second-line tisagenlecleucel, the cohort experienced decremental survival benefits of −0.02 QALYs at the incremental costs of $37 803 from the health care sector perspective and $39 480 from the societal perspective (Table 2). As a result, tisagenlecleucel was dominated by salvage chemotherapy followed by HSCT as a second-line therapy.

**Table 2.** Estimated Base-Case Cost and Utility Outcomes After Modeling Over the Lifetime Horizon

Analysis perspectives	Cost, $^a^	LYs	QALYs	Incremental		ICER per QALY, $^a^
				Costs, $^a^	QALYs	
**Base case**						
Health care sector perspectives						
Axicabtagene ciloleucel (2L)	678 903	8.01	4.53	59 754	0.60	99 101
Standard care	619 149	7.50	3.93	NA	NA	NA
Tisagenlecleucel (2L)	534 426	3.16	2.02	37 803	−0.02	Dominated^b^
Standard care	496 623	3.45	2.04	NA	NA	NA
Tisagenlecleucel (≥3L)	489 767	7.66	3.86	271 399	2.14	126 593
Standard care	218 368	3.20	1.72	NA	NA	NA
Societal perspectives						
Axicabtagene ciloleucel (2L)	688 507	8.01	4.55	59 076	0.60	97 977
Standard care	629 431	7.50	3.94	NA	NA	NA
Tisagenlecleucel (2L)	543 578	3.16	2.02	39 480	−0.02	Dominated^b^
Standard care	504 098	3.45	2.04	NA	NA	NA
Tisagenlecleucel (≥3L)	499 457	7.66	3.86	274 442	2.14	128 012
Standard care	225 016	3.20	1.72	NA	NA	NA
**No crossover scenario**						
Health care sector perspectives						
Axicabtagene ciloleucel (2L)	701 407	10.17	5.31	428 745	1.98	216 790
Standard care	272 661	7.36	3.33	NA	NA	NA
Societal perspectives						
Axicabtagene ciloleucel (2L)	710 450	10.17	5.31	432 933	1.98	218 907
Standard care	277 517	7.36	3.33	NA	NA	NA
**Crossover with improved progression-free survival scenario**						
Health care sector perspective						
Axicabtagene ciloleucel (2L)	671 496	8.01	4.53	75 950	0.60	125 962
Standard care	595 546	7.50	3.93	NA	NA	NA
Societal perspective						
Axicabtagene ciloleucel (2L)	681 391	8.01	4.53	74 122	0.60	122 931
Standard care	607 269	7.50	3.93	NA	NA	NA

Abbreviations: ICER, incremental cost-effectiveness ratio; L, line of therapy; LY, life-years; NA, not applicable; QALY, quality-adjusted life-years.

^a^ Costs are provided in adjusted 2021 US dollars.

^b^ One treatment is considered dominated by the other when it results in incremental costs but decremental effectiveness.

### Third-line or Later Tisagenlecleucel

In the third-line or later settings, the tisagenlecleucel group experienced incremental survival benefits of 2.14 QALYs, at the incremental expense of $271 399 from the health care sector perspective and $274 442 from the societal perspective vs the salvage-treatment group (Table 2). From the health care sector perspective, the ICER was $126 593 per QALY, and from the societal perspective, the ICER was $128 012 per QALY.

### Scenario Analyses

The scenario analysis of no treatment switching to CAR T cells after SC estimated incremental QALYs and incremental costs associated with axicabtagene ciloleucel compared with salvage chemotherapy followed by HSCT only (Table 2). The axicabtagene ciloleucel group experienced incremental survival benefits of 1.98 QALYs at the incremental costs of $428 745 from the health care sector perspective and $432 933 from the societal perspective, yielding an ICER of $216 790 per QALY from the health care sector perspective and an ICER of $218 907 per QALY from the societal perspective. The second scenario assumed that patients who did not response to SC and switched to third-line CAR T cell therapy had improved PFS and lower progression-related costs, yielding an ICER of $125 962 per QALY from the health care sector perspective and an ICER of $122 931 per QALY from the societal perspective (Table 2). The third scenario explored the impact of the treatment center administering the therapy and resulted in no significant difference in ICERs, implying a minimal impact of center type on the cost-effectiveness of CAR T cell therapy (eTable 3 in Supplement 1).

### Sensitivity Analyses

**Figure 2.**
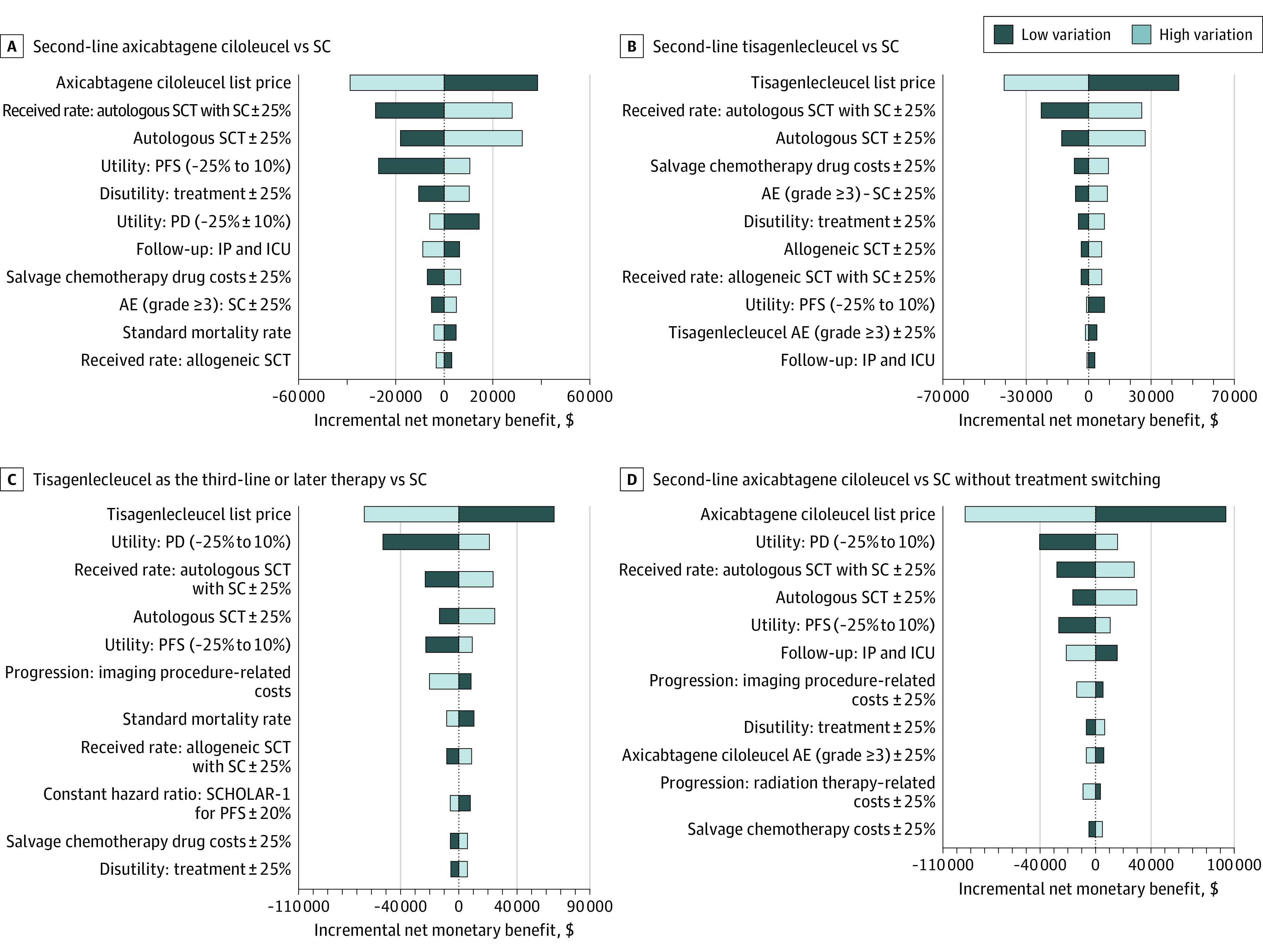
Univariate Sensitivity Analysis AE indicates adverse events; ICU, intensive care unit; IP, inpatient; PD, progressive disease; PFS, progression-free survival; SC, standard care; and SCT, stem cell transplantation.

Univariate sensitivity analyses indicated that ICERs from axicabtagene ciloleucel were consistently cost-effective at the $150 000 per QALY willingness-to-pay threshold under all parametric variations (Figure 2). Similarly, second-line tisagenlecleucel was either dominated by SC or was not cost-effective under all parametric variations. ICERs from third-line or later tisagenlecleucel were consistently cost-effective across the univariate sensitivity analyses; however, a decrease in utility from PD below 0.5 QALYs or an increase in tisagenlecleucel’s list price higher than $466 250 would surpass the cost-effectiveness threshold. Specific to third-line or later salvage treatment, we tested the effect of the constant hazard ratio approach to PFS on our calculations and did not find a difference from the base-case conclusion.

**Figure 3.**
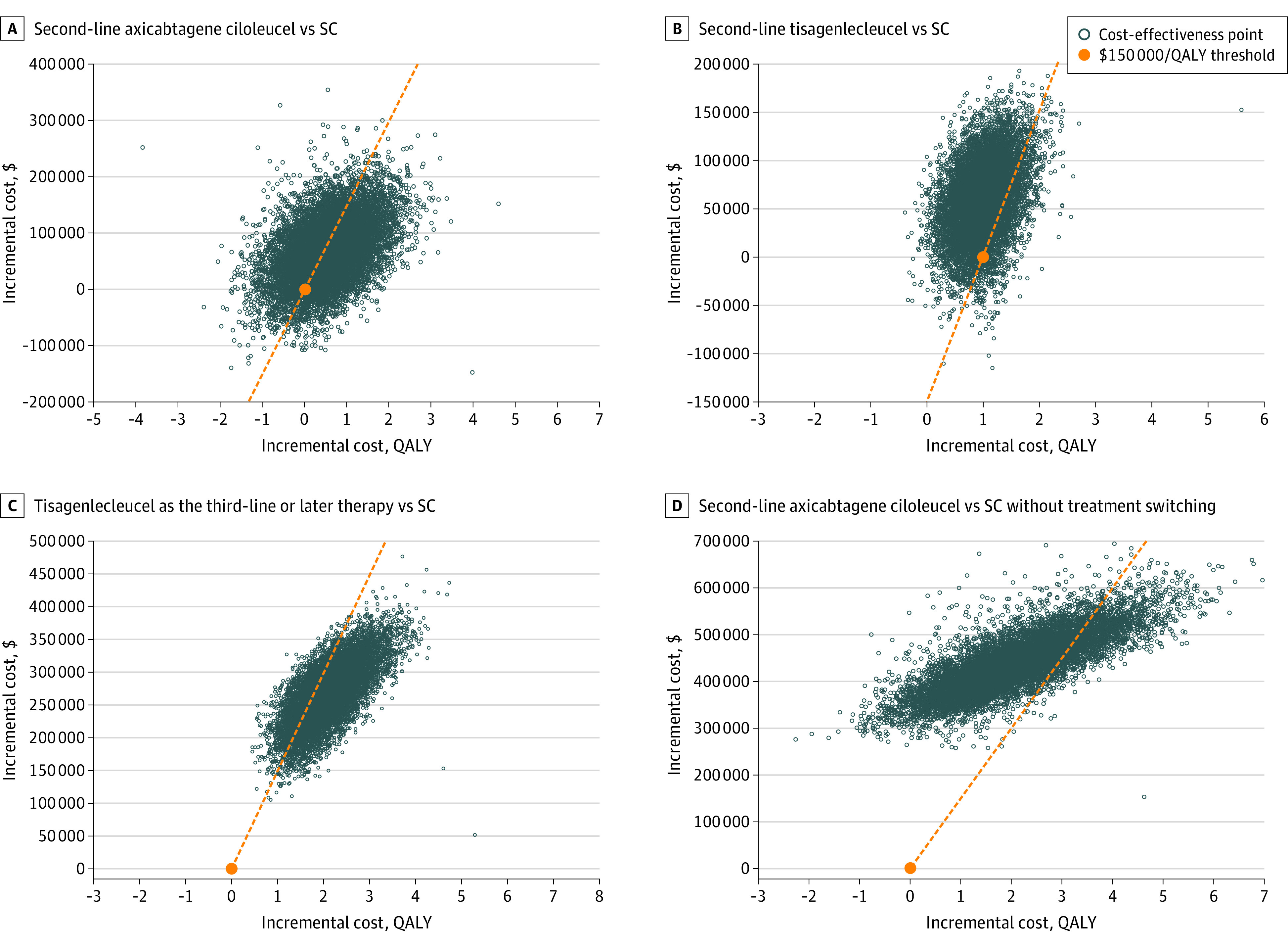
Scatterplot of Costs and Effectiveness Outcomes From a Probabilistic Sensitivity Analysis Through 10 000 Simulations QALY indicates quality-adjusted life-years; SC, standard care.

The probabilistic sensitivity analysis results with 10 000 Monte Carlo simulations were consistent with the base case ICERs. The probability of second-line axicabtagene ciloleucel being cost-effective was 44% at a willingness-to-pay threshold of $100 000 per QALY and 57% at a threshold of $150 000 per QALY (Figure 3). The probability of second-line tisagenlecleucel being cost-effective was 9% at the $100 000 per QALY threshold and 15% at the $150 000 per QALY threshold, and for third-line tisagenlecleucel, the probability of cost-effectiveness was 9% at the $100 000 per QALY threshold and 79% at the $150 000 per QALY threshold (Figure 3). Finally, based on the scenario analysis of no treatment switching to CAR T cells after SC, the probability of second-line axicabtagene ciloleucel being cost-effective was 0% at the $100 000 per QALY threshold and 17% at the $150 000 per QALY threshold (Figure 3). Nevertheless, the probabilistic sensitivity analysis demonstrated a mean incremental survival benefit of 2.10 QALYs when axicabtagene ciloleucel was compared with SC with no treatment switching based on the 10 000 simulations. Cost-effectiveness acceptability curves are presented in eFigure 3 in Supplement 1.

We also performed a threshold analysis of the CAR T cell therapy list price on the cost-effectiveness results from the US societal perspective. Our estimated value-based pricing for axicabtagene ciloleucel, compared with SC alone (without treatment switching) was $262 721, discounting approximately 34% from the current list price of $399 000 at the willingness-to-pay threshold of $150 000 per QALY. We applied this discounted price to our base case model. The analysis found that second-line axicabtagene ciloleucel was associated with incremental QALYs of 0.60 at the incremental costs of $6502, yielding an ICER of $10 783 per QALY. The probabilistic sensitivity analysis for the discounted axicabtagene ciloleucel price demonstrated a probability of cost-effectiveness of 75% at the $100 000 per QALY threshold and 78% at the $150 000 per QALY threshold.

The expected values of perfect information were estimated at $31 989 per patient for second-line axicabtagene ciloleucel, $4539 per patient for second line tisagenlecleucel, $6684 per patient for third-line or later tisagenlecleucel, and $12 936 per patient for the scenario analysis of no treatment switching. These estimates indicate that the cost per patient to obtain new information with greater certainty is not a barrier.^38,39^

## Discussion

This economic evaluation assessed axicabtagene ciloleucel as the second-line therapy and tisagenlecleucel as the second-line and third-line or later therapy in treating patients with relapsed or refractory DLBCL from the US health care sector and societal perspectives. We found second-line axicabtagene ciloleucel and third-line or later tisagenlecleucel to be cost-effective at the willingness-to-pay threshold of $150 000 per QALY, whereas second-line tisagenlecleucel was dominated by SC. Nevertheless, the probabilistic sensitivity analysis demonstrated a 57% probability that axicabtagene ciloleucel would be cost-effective at this threshold. Our probabilistic sensitivity analysis at the current list price for commercial CAR T cell therapy reflects the value of conducting research to identify patients who can achieve the best clinical outcomes or explore alternative pricing models for CAR T cell therapy.

Our study supports that tisagenlecleucel is cost-effective as a third-line or later therapy for patients with DLBCL but not as a second-line therapy. These results are aligned with BELINDA clinical outcomes, as second-line tisagenlecleucel did not meet the primary end point of EFS. Our probabilistic sensitivity analysis simulations showed that there were no survival benefits associated with second-line tisagenlecleucel vs SC. As a third-line or later therapy, the univariate sensitivity analysis confirmed that ICER for tisagenlecleucel remained cost-effective at the threshold of $150 000 per QALY within 25% variation of HSCT rates after CAR T cell therapy. We expect that a reduced list price of tisagenlecleucel would increase the confidence in its cost-effectiveness for most patients.

We conducted multiple scenario analyses, most importantly, to assess the cost-effectiveness of axicabtagene ciloleucel against SC in case of crossover from SC to CAR T cell therapy. Assuming that no crossover occurred, axicabtagene ciloleucel was associated with an ICER of $218 907 per QALY. Despite of the improved incremental survival gains in the axicabtagene ciloleucel group compared with SC without treatment switching, the incremental cost remains too high, rendering axicabtagene ciloleucel not cost-effective at the $150 000 per QALY threshold. This reflects the importance of price reduction as indicated earlier. The other scenario accounted for patients with no response in the SC group who received third-line CAR T cells and who would benefit from improved PFS and lower progression-associated costs. We adjusted the base-case model assuming these patients would progress into another round of PFS, PD, and death states. We used the same approach as in the base case to estimate total QALY gains, which seemed fitting since the JULIET trial provided the utility of CAR T cell therapy as a third-line treatment. We were able to calculate progression-related costs based on the retrospective US Lymphoma CAR T Consortium study.^37^ The findings of the scenario analysis suggest that second-line axicabtagene ciloleucel remained cost-effective but only at the $150 000 per QALY threshold. Thus, depending on the willingness-to-pay threshold, second-line axicabtagene ciloleucel might be overpriced.

To our knowledge, this is the first study to evaluate the cost-effectiveness of second-line CAR T cell therapy vs salvage chemotherapy followed by HSCT in patients with relapsed or refractory DLBCL from the US societal perspective. This perspective entails that the cost-effectiveness analysis measures both health-related and non–health-related costs caused by the intervention. This was achieved by incorporating travel-related indirect costs as part of a patients’ decision-making process into the base-case models. We also approached the models assuming that patients with relapsed or refractory DLBCL were attracted to the 1-time treatment and curative potential of CAR T cell therapies. Interestingly, the results of adapting a societal perspective did not differ significantly from those achieved by a health care payer perspective, probably owing to the high costs of CAR T cell therapy.

In 2022, two other reports have been published suggesting that second-line axicabtagene ciloleucel would be cost-effective from US health care sector perspective.^40,41^ Perales and colleagues^41^ used a mixture of cure models to extrapolate survival outcomes from axicabtagene ciloleucel as reported in ZUMA-7 and concluded that second-line axicabtagene ciloleucel is a cost-effective treatment, with an ICER of $66 381 per QALY. Similarly, Kambhampati and colleagues^40^ concluded that second-line axicabtagene ciloleucel is cost-effective, with an ICER of $93 547 per QALY. The study by Perales et al^41^ did not account for progression-related costs associated with CAR T cell crossover, while the study by Kambhampati et al^40^ modeled these outcomes based on the ZUMA-1 study. Our findings aligned with these results, although the standard parametric modeling approach that we used yields fewer mean survival gains than the mixture cure modeling approach and is considered more conservative.^11^

We explored the impact of uncertainties in clinical outcomes, utilities, and costs on the cost-effectiveness results by conducting probabilistic sensitivity analysis over 10 000 simulations. Based on our expected value of perfect information outcomes, when all these uncertainties are removed (ie, identifying the best treatment option for an individual patient), patients in the US are projected to save a total of $291 million when eligible patients with DLBCL receive second-line treatment and $21 million when eligible patients receive third-line or later treatment.^42^ Some of the uncertainties related to clinical outcomes of CAR T cell therapy can be associated with different bridging therapies and lymphodepletion approaches administer to prepare patients for infusion.^43^ As more CAR T cell therapies reach the market for a variety of indications, further studies on optimal treatment strategies for patients receiving CAR T cells are imperative. Most importantly, future research should focus on performing head-to-head trials comparing CAR T cells with SC to generate robust clinical evidence.

### Limitations

Our analysis may be limited by several factors. First, the total costs may be underestimated due to the lack of complete data sets on costs. Owing to scarcity of information on utilization and treatment costs of CAR T cells in practice, we adopted the estimated tisagenlecleucel costs from the JULIET trial based on the US hospital perspective in clinical trial settings, which may differ from practice settings.^22^ Additionally, out-of-pocket expenses other than travel-related opportunity costs were not included due to lack of data. Second, utilities for both second- and third-line therapy were extrapolated from the third-line or later tisagenlecleucel clinical trial due to absence of utilities for second-line therapy. Third, the PFS curve for SCHOLAR-1 may follow a different parametric curve than the OS curve. Although we tested the univariate sensitivity analysis on the constant hazard ratio and the results were robust under the variations, the true PFS curve may reveal different results.

## Conclusions

The findings of this economic evaluation suggest that second-line axicabtagene ciloleucel and third-line or later tisagenlecleucel were cost-effective compared with the salvage chemotherapy followed by HSCT in patients with relapsed or refractory DLBCL from both the US health care sector and societal perspectives at the willingness-to-pay threshold of $150 000 per QALY. Our study underscores the importance of additional research to ensure the delivery of CAR T cell therapy to the right patient at the right time so that the drug price is aligned with patient benefits.

## Notes

Supplement 1.
eMethods.
**eFigure 1.** Visual Fits of Standard Parametric Functions **eFigure 2.** Extended Parametric Survival Curves for CAR T Cell Therapies and Standard Care **eTable 1.** Statistical Fits for Parametric Modeling **eTable 2.** Calculation of Adverse Events **eTable 3.** Results From Scenario Analysis on Type of CAR T Cell Therapy Treatment Center **eFigure 3.** Cost-Effectiveness Acceptability Curves
eReferences.

Supplement 2.
Data Sharing Statement
