# Supplementary material for: Cost-effectiveness of Axicabtagene Ciloleucel and Tisagenlecleucel as Second-line or Later Therapy in Relapsed or Refractory Diffuse Large B-Cell Lymphoma
Source: JAMA Netw Open. 2022 Dec 15;5(12):e2245956. doi: 10.1001/jamanetworkopen.2022.45956 (PMC9856352; doi:10.1001/jamanetworkopen.2022.45956)
Supplement: Supplement 1. — eMethods. eFigure 1. Visual Fits of Standard Parametric Functions eFigure 2. Extended Parametric Survival Curves for CAR T Cell Therapies and Standard Care eTable 1. Statistical Fits for Parametric Modeling eTable 2. Calculation of Adverse Events eTable 3. Results From Scenario Analysis on Type of CAR T Cell Therapy Treatment Center eFigure 3. Cost-Effectiveness Acceptability Curves eReferences. [file jamanetwopen-e2245956-s001.pdf]

Supplemental Online Content

Choe JH, Abdel-Azim H, Padula WV, Abou-el-Enein M. Cost-effectiveness of axicabtagene ciloleucel and tisagenlecleucel as second-line or later therapy in relapsed or refractory diffuse large B-cell lymphoma. *JAMA Netw Open*. 2022;5(12):e2245956. doi:10.1001/jamanetworkopen.2022.45956

eMethods.
eFigure 1. Visual Fits of Standard Parametric Functions
eFigure 2. Extended Parametric Survival Curves for CAR T Cell Therapies and Standard Care
eTable 1. Statistical Fits for Parametric Modeling
eTable 2. Calculation of Adverse Events
eTable 3. Results From Scenario Analysis on Type of CAR T Cell Therapy Treatment Center
eFigure 3. Cost-Effectiveness Acceptability Curves
eReferences.

This supplemental material has been provided by the authors to give readers additional information about their work.

© 2022 Choe JH et al. *JAMA Network Open.*

eMethods.

Technical Appendix
1. Assumptions

A. Based on the plateau achieved around 40 months from the JULIET trial, we assumed the curative potential of chimeric antigen receptor (CAR) T cell therapy and HSCT at 40 months (1).
B. There is a potential confounding effect of crossover from the standard care to CAR T cell therapy on overall survival (OS) curves. The scenario analysis was conducted to axi-cel versus salvage chemotherapy followed by HSCT only by extrapolating transitional probabilities from sensitivity analysis of OS using the Rank Preserving Structural Failure Time method (2).
C. As the PFS curve was not available from SCHOLAR-1, we hypothesized that the progression-free survival (PFS) curve will follow the OS curve with the constant hazard ratio of 0.7. The assumption is tested by the one-way sensitivity analysis.
D. We included grade 3 or above adverse events with incidence rates greater than 5%.
E. Because SCHOLAR-1 did not report the safety profile, we used the adverse events reported on one of the included trials for SCHOLAR-1 (3).
F. We directly used the utilities from PFS and PD from the JULIET trial for all cohorts. One-way sensitivity analysis tested the impact of the utilities. We also assumed that patients who proceeded to third-line CAR T cell therapy would experience the same utility from PD state.

2. CEA Model Structure

Hypothetical US cohorts were referenced from the clinical trials. Both CAR T cell and standard-care groups were assumed to share the same age, proportion of male, and body surface area. Second-line axi-cel cohorts were 58 years old and 66% male (2); second-line tisa-cel cohorts were 58 years old and 60% male (4); third-line or above tisa-cel cohorts were 56 years old and 60% male (1). Additionally, the body surface area was assumed to be 1.92 m^2 (5).

Our model follows the guideline outlined by the Second Panel on Cost-Effectiveness in Health and Medicine (6). We chose the societal perspective as the reference case and also reported some of the analyses from the healthcare perspective. The model's time horizon was a lifetime horizon, assuming the patient's expected age is 100 years old (7).

3. Salvage chemotherapy used as optional bridging therapy or standard of care

ZUMA-7 reported that salvage chemotherapies were R-ICE (rituximab, etoposide, ifosfamide, carboplatin, mesna), R-GDP (rituximab, gemcitabine, dexamethasone, cisplatin), R-DHAP (rituximab, dexamethasone, cisplatin, cytarabine), and R-ESHAP (rituximab, etoposide, methylprednisolone, cisplatin, cytarabine). Also, as optional bridging therapy was restricted to glucocorticoids only, we used dexamethasone (40 mg) as optional bridging for the second line axi-cel group (2).

BELINDA reported that salvage chemotherapies were R-ICE, R-GDP, R-DHAP, R-GemOX (rituximab, gemcitabine, oxaliplatin), and optional bridging therapy for the second line tisa-cel group would be one of the four above chemotherapies. We assumed an equal probability of receiving any bridging options (4).

Salvage chemotherapies were R-ICE, R-GDP, R-DHAP, and R-GemOX for the standard-care group in the third-line or above setting (8). Since JULIET did not report optional bridging therapy, we assumed patients received ifosfamide-etoposide (50%), ifosfamide-etoposide-mitoxantr (21%), and cyclophosphamide, doxorubicin, vincristine, prednisone (CHOP, 29%) (9).

4. Validation of Parametric Survival Functions

National Institute for Health and Care Excellence (NICE) recommended using parametric survival functions derived from individual-patient level data (IPD) to accurately estimate the survival benefit (10). However, in the absence of IPD, we calibrated parametric survival functions by using R packages (11). Firstly, Kaplan-Meier (KM) curves (OS and PFS) were digitalized by using WebPlotDigitizer.com (WebPlotDigitizer, version 4.5). Then, we used R packages to estimate the IPD points (R, version 4.1.3) and cross-checked the similarity between the actual KM graph and the estimated KM graph. With IPD points, we run regression analysis by using Stata (Stata, version 17). Finally, following the NICE document, we compared Akaike's Information Criterion (AIC) values. The best-fit parametric survival function was chosen based on the lowest sum of AIC and BIC values (eTable 1). However, when the selected survival function with the lowest does not align with the published KM curve, then we chose the parametric function based on the lowest AIC or BIC and visualizations. Below figures and tables are validation of our parametric functions.

© 2022 Choe JH et al. *JAMA Network Open.*

eFigure 1. Visual Fits of Standard Parametric Functions

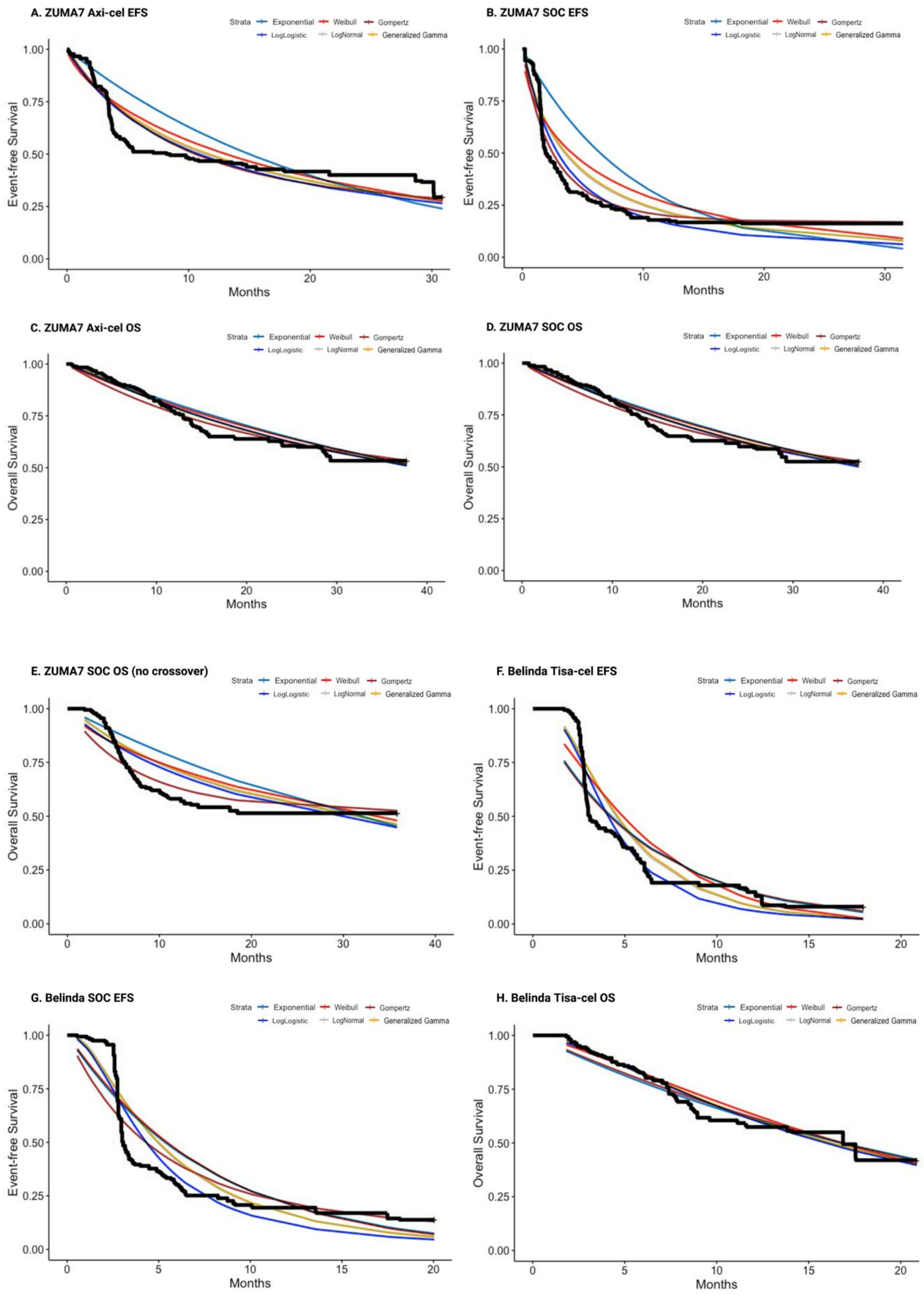

© 2022 Choe JH et al. *JAMA Network Open.*

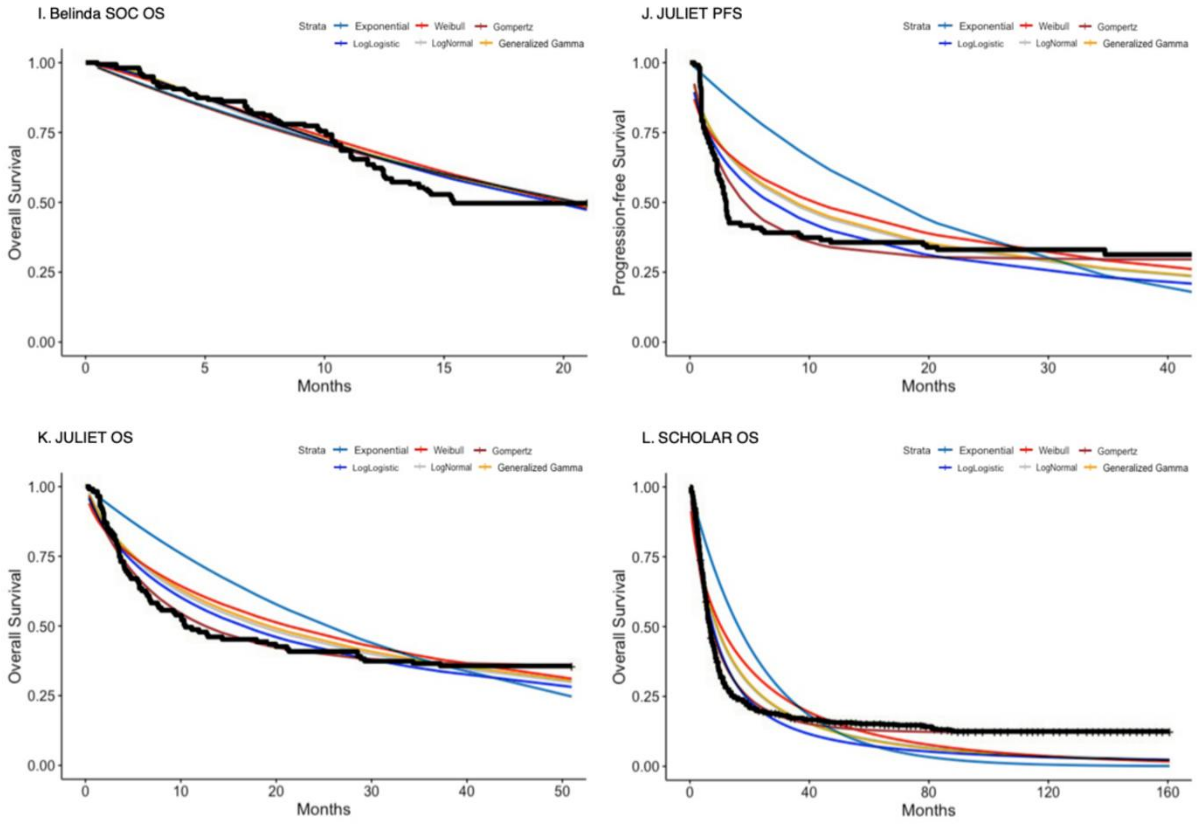

axi-cel; axicabtagene ciloleucel; EFS, event-free survival; PFS, progression-free survival; OS, overall survival; SOC, standard of care; tisa-cel, tisagenlecleucel.

© 2022 Choe JH et al. *JAMA Network Open.*

eFigure 2. Extended Parametric Survival Curves for CAR T Cell Therapies and Standard Care

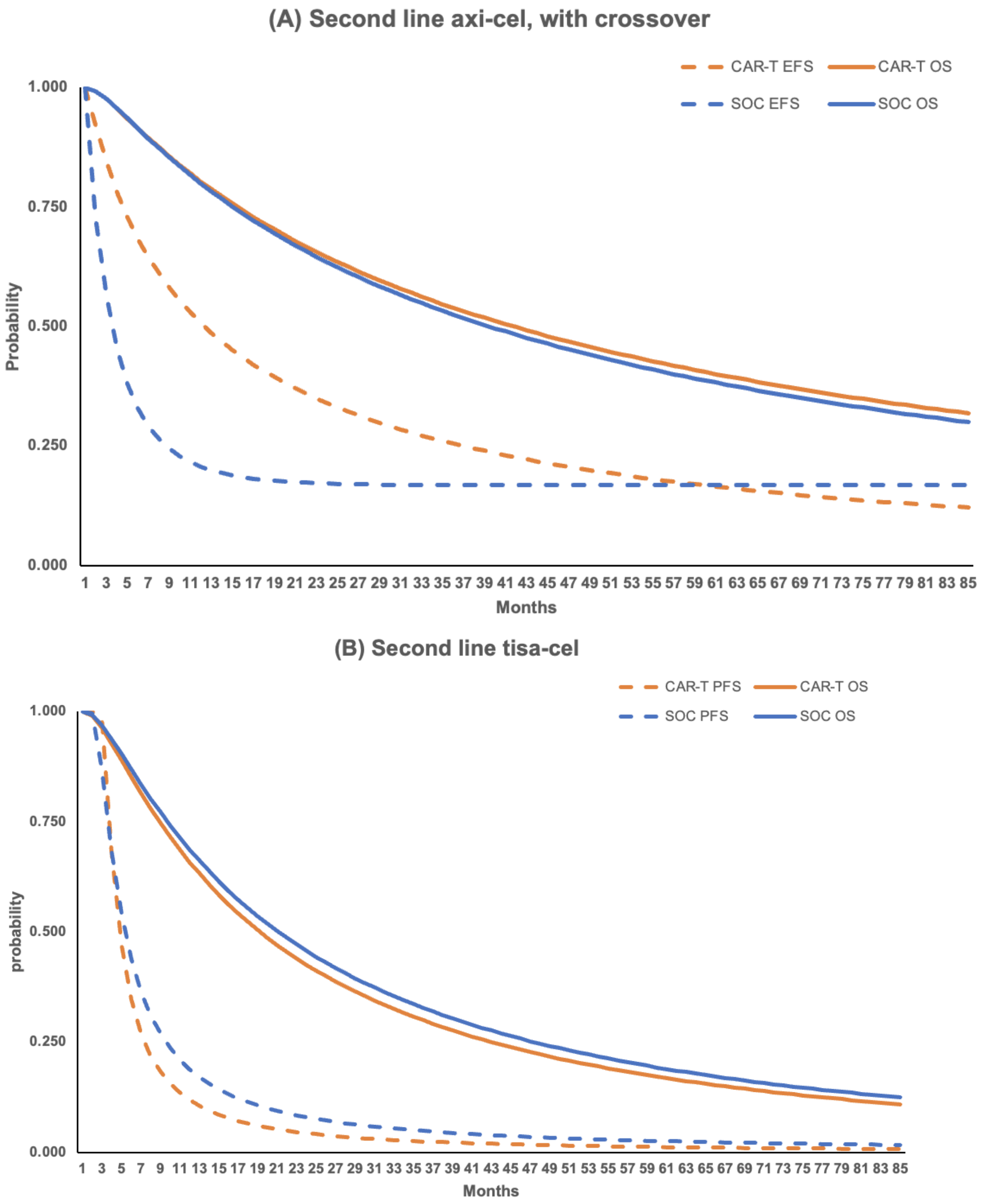

© 2022 Choe JH et al. *JAMA Network Open.*

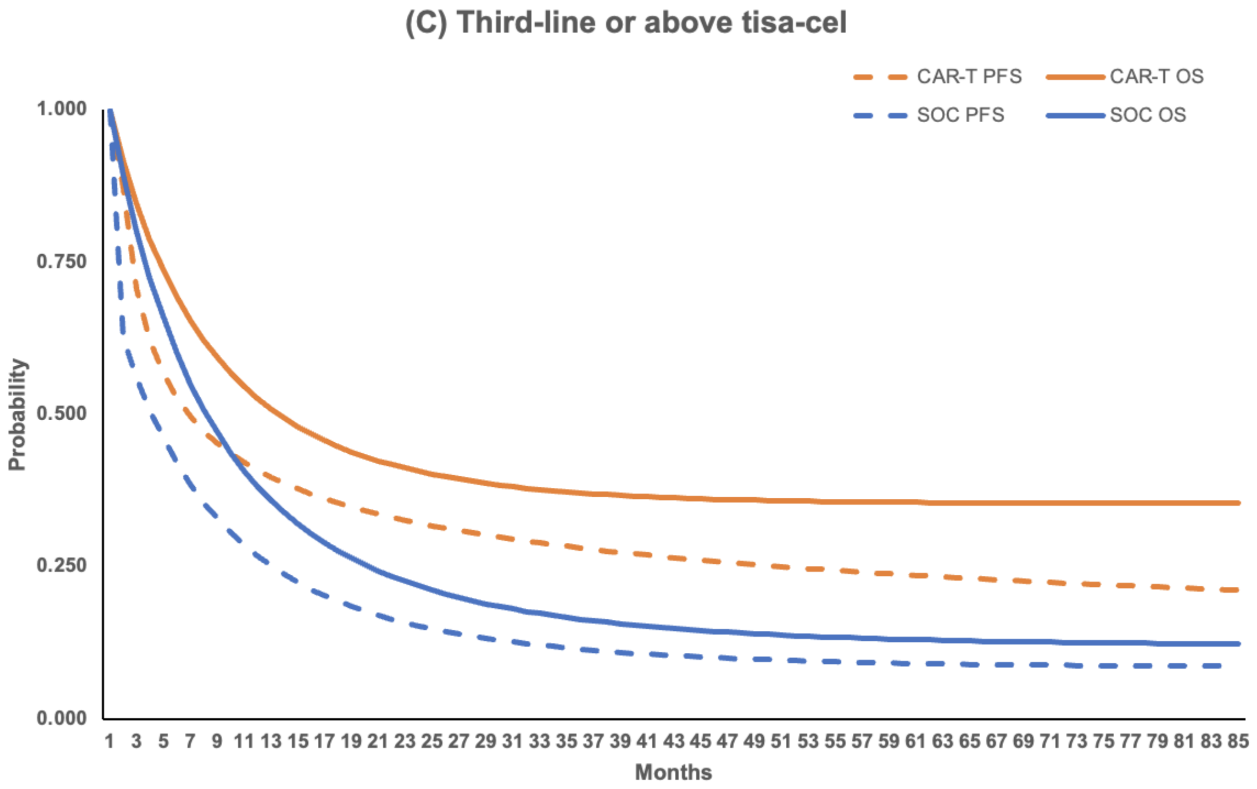

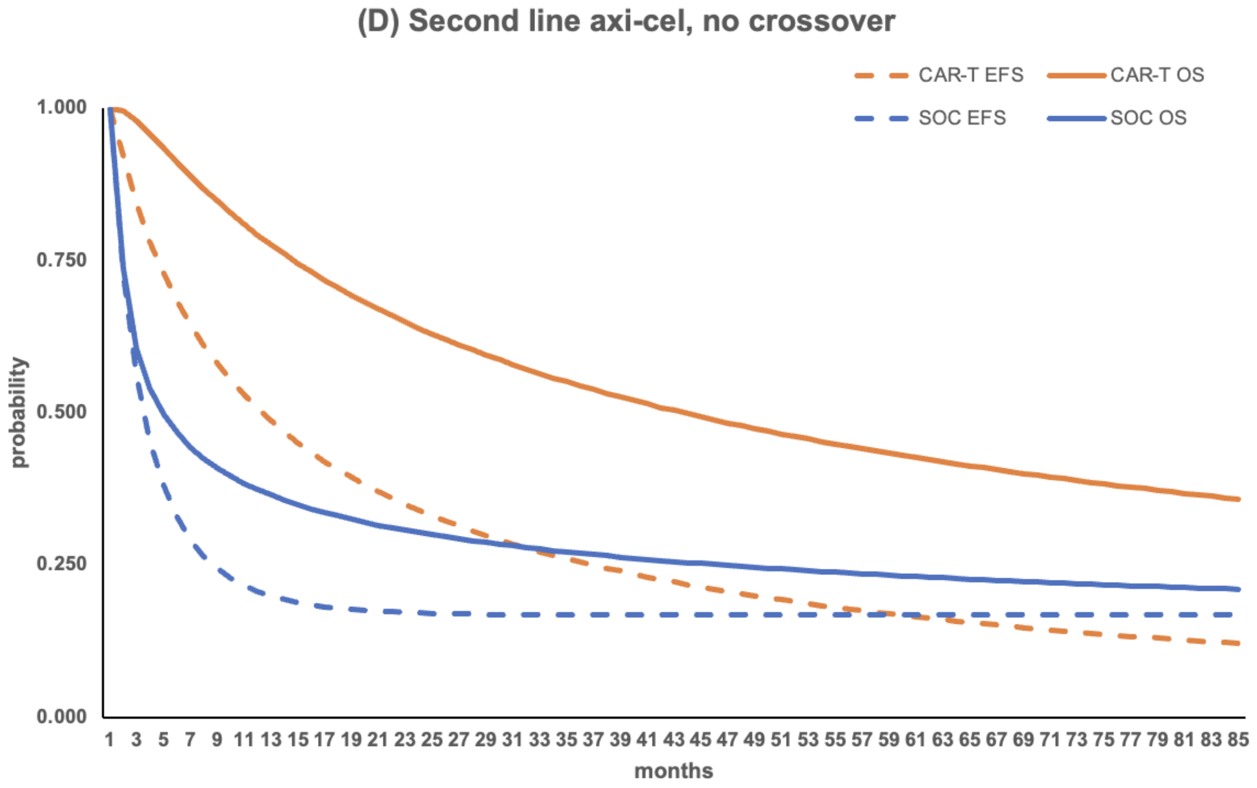

Axi-cel, axicabtagene ciloleucel; CAR-T, chimeric antigen receptor t-cell therapy; EFS, event-free survival; PFS, progression-free survival; OS, overall survival; tisa-cel, tisagenlecleucel; SOC, standard of care.

© 2022 Choe JH et al. *JAMA Network Open.*

eTable 1. Statistical Fits for Parametric Modeling

Parametric Function	ZUMA-7 OS						ZUMA-7 EFS			
	Axi-cel		Salvage Chemo/HSCT ± CAR-T		Salvage chemo/HSCT		Axi-cel		Salvage chemo/HSCT	
	AIC	BIC	AIC	BIC	AIC	BIC	AIC	BIC	AIC	BIC
Exponential	451.52	454.72	448.66	451.85	513.62	516.80	638.20	641.39	765.61	768.80
Weibull	453.20	459.58	450.53	456.89	503.65	510.03	618.05	624.44	692.91	699.28
Gompertz	447.01	453.40	445.04	451.41	452.49	458.86	608.05	614.43	**583.69**	**590.07**
Log-Logistics	447.19	453.58	444.25	450.61	488.15	494.53	603.97	610.35	624.19	630.56
Log-Normal	**443.47**	**449.85**	**440.46**	**446.82**	477.06	483.43	598.55	604.94	632.53	638.90
Generalized Gamma	443.96	453.54	440.75	450.30	**416.50**	**426.06**	**597.55**	607.13	600.66	610.22

Parametric Function	BELINDA OS				BELINDA EFS			
	Tisa-cel		Salvage Chemo/HSCT ± CAR-T		Tisa-cel		Salvage chemo/HSCT	
	AIC	BIC	AIC	BIC	AIC	BIC	AIC	BIC
Exponential	408.67	411.75	384.07	387.14	433.93	437.01	477.50	480.57
Weibull	405.73	411.90	383.79	389.93	421.31	427.48	479.30	485.43
Gompertz	410.35	416.53	386.03	392.17	435.79	441.97	463.94	470.08
Log-Logistics	398.93	405.10	378.45	384.58	350.88	357.06	415.18	421.32
Log-Normal	**393.68**	**399.86**	**377.02**	**383.16**	356.25	362.43	420.92	427.06
Generalized Gamma	390.93	400.19	378.84	388.05	**264.09**	**273.35**	**379.41**	**388.62**

© 2022 Choe JH et al. *JAMA Network Open.*

Parametric Function	JULIET & SCHOLAR OS				JULIET PFS	
	Tisa-cel ± HSCT		Salvage Chemo/HSCT		Tisa-cel ± HSCT	
	AIC	BIC	AIC	BIC	AIC	BIC
Exponential	438.01	440.76	2505.40	2509.81	539.87	542.61
Weibull	406.96	412.45	2273.28	2282.08	454.69	460.18
Gompertz	368.76	374.25	1988.33	1997.14	388.56	394.05
Log-Logistics	391.95	397.44	2038.66	2047.46	430.14	435.63
Log-Normal	386.53	392.02	2066.77	2075.57	425.14	430.63
Generalized Gamma	**366.46**	**374.69**	**1967.66**	**1980.87**	**364.53**	**372.76**

Axi-cel, axicabtagene ciloleucel; chemo, chemotherapy; CAR-T, chimeric antigen receptor T-cell therapy; EFS, event-free survival; HSCT, hematopoietic stem-cell transplantation; PFS, progression-free survival; OS, overall survival; tisa-cel, tisagenlecleucel

© 2022 Choe JH et al. *JAMA Network Open.*

eTable 2. Calculation of Adverse Events

A. Axi-cel vs Standard care as second-line therapy in r/r DLBCL

**Adverse Events (Grade 3/4)**	**Axi-cel %**	**Standard-Care %**	**Cost per event**	**Source**
Anemia	30%	39%	$9,501.79	HCUP (12)
Thrombocytopenia	15%	57%	$12,562.64	HCUP (12)
Neutropenia	69%	41%	$13,357.01	HCUP (12)
CRS	6%	0%	$20,375.39	HCUP (12)
Hypokalemia	6%	7%	$7,027.44	HCUP (12)
Pyrexia	9%	0%	$6,549.00	HCUP (13)
Fatigue	6%	0%	$11,015.24	HCUP (12)
Febrile neutropenia	0%	13%	$19,984.81	HCUP (12)
Leukopenia	29%	22%	$7,890.00	HCUP (13)
Hypophosphatemia	18%	12%	$7,345.62	HCUP (12)
Hypoxia	9%	4%	$16,409.26	HCUP (14)
Neurological Events	21%	0%	$14,846.00	Broder et al. (15)
Hypotension	11%	0%	$6,853.00	HCUP (12)

B. Tisa-cel vs Standard care as second-line therapy in r/r DLBCL

**Adverse Events (Grade 3/4)**	**Tisa-cel %**	**Standard-Care %**	**Cost per event**	**Source**
Anemia	33%	58%	$9,501.79	HCUP (12)
Nausea	1%	6%	$7,066.33	HCUP (12)
Thrombocytopenia	32%	48%	$12,562.64	HCUP (12)
Neutropenia	40%	39%	$13,357.01	HCUP (12)
CRS	5%	0%	$20,375.39	HCUP (12)
Hypokalemia	5%	9%	$7,027.44	HCUP (12)
Platelet count decrease	20%	31%	$12,562.64	HCUP (12)
Neutrophil count decrease	25%	19%	$13,357.01	HCUP (12)
Febrile neutropenia	13%	25%	$19,984.81	HCUP (12)
Leukopenia	13%	18%	$7,890.00	HCUP (13)
WBC count decrease	11%	12%	$8,063.63	HCUP (12)

© 2022 Choe JH et al. *JAMA Network Open.*

C. SCHOLAR-1 based on Crump 2014[3]

**Adverse Events (Grade 3/4)**	**Salvage Treatment %**	**Cost per event**	**Source**
Thrombosis/embolism	6%	$9,100.00	HCUP (16)
Fatigue	10%	$11,015.24	HCUP (12)
Nausea	6%	$7,066.33	HCUP (14)
Vomiting	7%	$7,066.33	HCUP (14)
Grade 3 to 5 Neutropenia	8%	$13,357.01	HCUP (12)
Without Neutropenia	7%	$8,254.61	HCUP (14)
Febrile neutropenia	16%	$19,984.81	HCUP (12)

DLBCL, diffuse large B-cell lymphoma; r/r, relapsed or refractory.

© 2022 Choe JH et al. *JAMA Network Open.*

eTable 3. Results From Scenario Analysis on Type of CAR T Cell Therapy Treatment Center

Analysis Perspective		Cost, $	LYs	QALYs	Incremental Costs	Incremental QALYs	ICER per QALY
1. Academic centers only							
	Axi-cel (2L)	$690,196	8.01	4.53	$60,287	0.60	$99,986
	Standard Care	$629,909	7.50	3.93			
	Tisa-cel (2L)	$545,251	3.16	2.02	$41,153	-0.02	Dominated
	Standard Care	$504,098	3.45	2.04			
	Tisa-cel (3L+)	$500,901	7.66	3.86	$275,885	2.14	$128,685
	Standard Care	$225,016	3.20	1.72			
2. Any Specialties							
	Axi-cel (2L)	$686,978	8.01	4.53	$57,979	0.60	$96,158
	Standard Care	$628,999	7.50	3.93			
	Tisa-cel (2L)	$542,063	3.16	2.02	$37,965	-0.02	Dominated
	Standard Care	$504,098	3.45	2.04			
	Tisa-cel (3L+)	$498,151	7.66	3.86	$273,135	2.14	$127,403
	Standard Care	$225,016	3.20	1.72			

Axi-cel, axicabtagene ciloleucel; 2L, second-line therapy; 3L+, third-line or above therapy; CAR-T, chimeric antigen receptor T-cell therapy; ICER, incremental cost-effectiveness ratio; LY, life year; QALY, quality-adjusted life-years; tisa-cel, tisagenlecleucel

© 2022 Choe JH et al. *JAMA Network Open.*

eFigure 3. Cost-Effectiveness Acceptability Curves

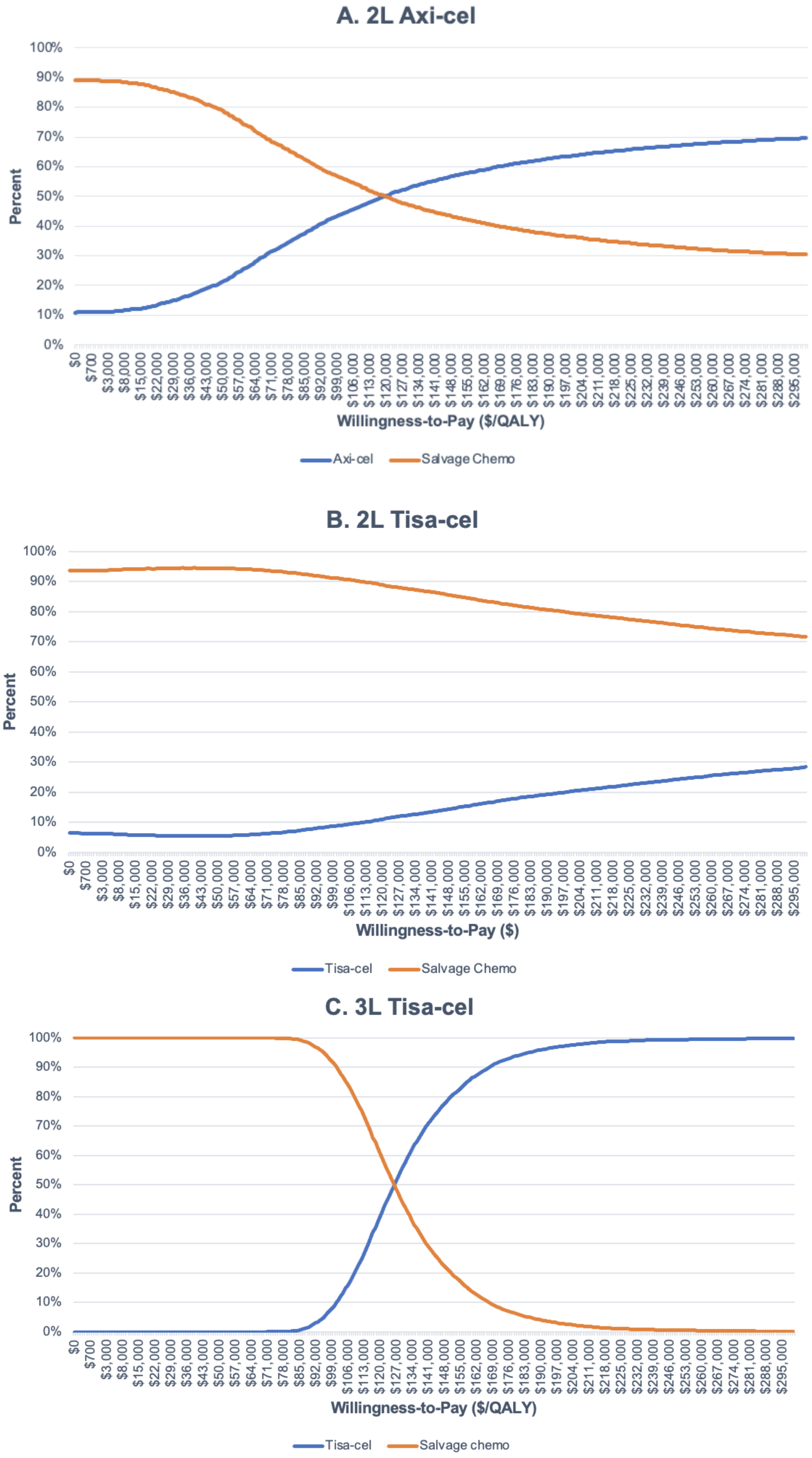

© 2022 Choe JH et al. *JAMA Network Open.*

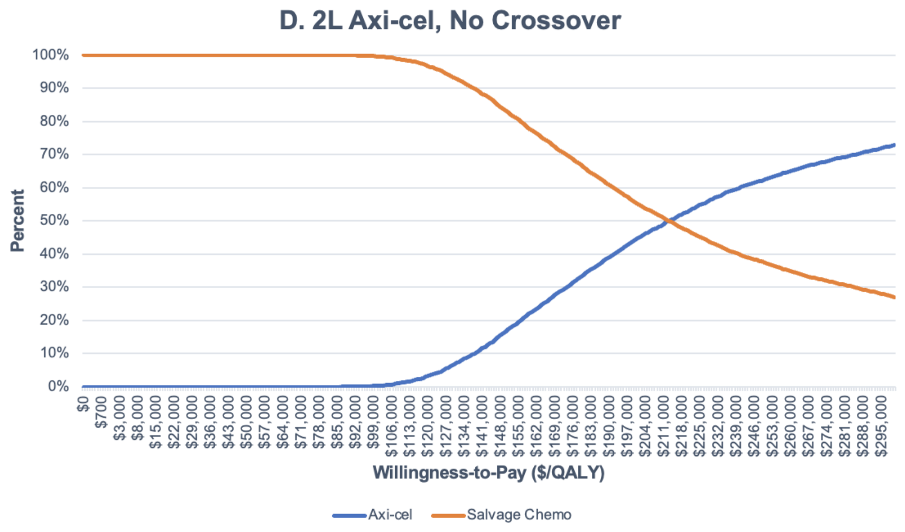

2L, second line; 3L, third-line or above; axi-cel, axicabtagene ciloleucel; tisa-cel, tisagenlecleucel.

© 2022 Choe JH et al. *JAMA Network Open.*

eReferences

1. Schuster SJ, Tam CS, Borchmann P, Worel N, McGuirk JP, Holte H, et al. Long-term clinical outcomes of tisagenlecleucel in patients with relapsed or refractory aggressive B-cell lymphomas (JULIET): a multicentre, open-label, single-arm, phase 2 study. Lancet Oncol. 2021 Oct;22(10):1403–15.

2. Locke FL, Miklos DB, Jacobson CA, Perales MA, Kersten MJ, Oluwole OO, et al. Axicabtagene Ciloleucel as Second-Line Therapy for Large B-Cell Lymphoma. N Engl J Med. 2022 Feb 17;386(7):640–54.

3. Crump M, Kuruvilla J, Couban S, MacDonald DA, Kukreti V, Kouroukis CT, et al. Randomized comparison of gemcitabine, dexamethasone, and cisplatin versus dexamethasone, cytarabine, and cisplatin chemotherapy before autologous stem-cell transplantation for relapsed and refractory aggressive lymphomas: NCIC-CTG LY.12. J Clin Oncol Off J Am Soc Clin Oncol. 2014 Nov 1;32(31):3490–6.

4. Bishop MR, Dickinson M, Purtill D, Barba P, Santoro A, Hamad N, et al. Second-Line Tisagenlecleucel or Standard Care in Aggressive B-Cell Lymphoma. N Engl J Med. 2022 Feb 17;386(7):629–39.

5. Canadian Agency for Drugs and Technologies in Health (CADTH). Tisagenlecleucel for Diffuse Large B-cell Lymphoma: Economic Review Report. CADTH; 2019 Jan. (CADTH optimal use report; vol.8, no.3e) [Internet]. [cited 2021 Aug 2]. Available from: https://cadth.ca/tisagenlecleucelacute-lymphoblastic-leukemia-and-diffuse-large-b-cell-lymphoma-recommendations

6. Neumann P, Sanders G, Russell L, Siegel J, Ganiats T. Cost-Effectiveness in Health and Medicine. Second edition. oxford University Press; 2017.

7. Gye A, Goodall S, De Abreu Lourenco R. A Systematic Review of Health Technology Assessments of Chimeric Antigen Receptor T-Cell Therapies in Young Compared With Older Patients. Value Health J Int Soc Pharmacoeconomics Outcomes Res. 2022 Jan;25(1):47–58.

8. Crump M, Neelapu SS, Farooq U, Van Den Neste E, Kuruvilla J, Westin J, et al. Outcomes in refractory diffuse large B-cell lymphoma: results from the international SCHOLAR-1 study. Blood. 2017 Oct 19;130(16):1800–8.

9. Sesques P, Ferrant E, Safar V, Wallet F, Tordo J, Dhomps A, et al. Commercial anti-CD19 CAR T cell therapy for patients with relapsed/refractory aggressive B cell lymphoma in a European center. Am J Hematol. 2020 Nov;95(11):1324–33.

10. Latimer N. NICE DSU Technical Support Document 14: Undertaking survival analysis for economic evaluations alongside clinical trials - extrapolation with patient-level data [Internet]. 2011. Available from: http://www.nicedsu.org.uk

11. Liu N, Zhou Y, Lee JJ. IPDfromKM: reconstruct individual patient data from published Kaplan-Meier survival curves. BMC Med Res Methodol. 2021 Jun 1;21(1):111.

12. Yang H, Hao Y, Chai X, Qi CZ, Wu EQ. Estimation of total costs in patients with relapsed or refractory diffuse large B-cell lymphoma receiving tisagenlecleucel from a US hospital's perspective. J Med Econ. 2020 Sep;23(9):1016–24.

13. Roth JA, Sullivan SD, Lin VW, Bansal A, Purdum AG, Navale L, et al. Cost-effectiveness of axicabtagene ciloleucel for adult patients with relapsed or refractory large B-cell lymphoma in the United States. J Med Econ. 2018 Dec;21(12):1238–45.

14. Agency for Healthcare Research and Quality (AHRQ). Healthcare Cost and Utilization Project (HCUP) Inpatient Database (2018). https://hcupnet.ahrq.gov/#setup. Accessed on November 29, 2021.

© 2022 Choe JH et al. *JAMA Network Open.*

15. Broder MS, Ma Q, Yan T, Zhang J, Chang E, Kuzan D, et al. Economic Burden of Neurologic Toxicities Associated with Treatment of Patients with Relapsed or Refractory Diffuse Large B-Cell Lymphoma in the United States. Am Health Drug Benefits. 2020 Nov;13(5):192–9.

16. Lin JK, Muffly LS, Spinner MA, Barnes JI, Owens DK, Goldhaber-Fiebert JD. Cost Effectiveness of Chimeric Antigen Receptor T-Cell Therapy in Multiply Relapsed or Refractory Adult Large B-Cell Lymphoma. J Clin Oncol Off J Am Soc Clin Oncol. 2019 Aug 20;37(24):2105–19.

© 2022 Choe JH et al. *JAMA Network Open.*
