# Supplementary material for: Cost-effectiveness of Axicabtagene Ciloleucel and Tisagenlecleucel as Second-line or Later Therapy in Relapsed or Refractory Diffuse Large B-Cell Lymphoma
Source: JAMA Netw Open. 2022 Dec 15;5(12):e2245956. doi: 10.1001/jamanetworkopen.2022.45956 (PMC9856352; doi:10.1001/jamanetworkopen.2022.45956)
Supplement: Supplement 2. — Data Sharing Statement [file jamanetwopen-e2245956-s002.pdf]

Data Sharing Statement

Choe. Cost-Effectiveness of Axicabtagene Ciloleucel and Tisagenlecleucel as Second-Line or Later Therapy in Relapsed or Refractory Diffuse Large B-Cell Lymphoma. *JAMA Netw Open*. Published December 15, 2022. doi:10.1001/jamanetworkopen.2022.45956

Data
Data available: Yes
Data types: Data (not involving human participants)
How to access data: JHC and MA had full access to all the data in the study and take responsibility for the integrity of the data and the accuracy of the data analysis. Emails: Mohamed.Abouelenein@med.usc.edu and jhchoe@usc.edu
When available: With publication

Supporting Documents
Document types: None

Additional Information
Who can access the data: Researchers whose proposed use of the data has been approved
Types of analyses: Cost-effectiveness analysis
Mechanisms of data availability: After approval of a proposal
